# Buschke-Löwenstein tumor with squamous cell carcinoma treated with chemo-radiation therapy and local surgical excision: report of three cases

**DOI:** 10.1186/1477-7819-11-231

**Published:** 2013-09-16

**Authors:** Marileda Indinnimeo, Alessio Impagnatiello, Gabriella D’Ettorre, Gloria Bernardi, Cosima Maria Moschella, Paolo Gozzo, Antonio Ciardi, Caterina Bangrazi, Francesca De Felice, Daniela Musio, Vincenzo Tombolini

**Affiliations:** 1 Department of Surgery Pietro Valdoni - UOD of Surgery and Rehabilitation of the Pelvic Floor, Umberto I Policlinic of Rome, Sapienza University of Rome, Via Giovanni Maria Lancisi, Roma 2 00161, Italy; 2 Department of Radiological, Oncological and Anatomic-Pathological Science - UOC of Pathological Anatomy and Histology, Umberto I Policlinic of Rome, Sapienza University of Rome, Via Giovanni Maria Lancisi, Roma 2 00161, Italy; 3 Department of Public Health and Infectious Diseases - UOC of Infectious Diseases, Umberto I Policlinic of Rome, Sapienza University of Rome, Viale Regina Elena, Roma 324 00161, Italy; 4 Department of Radiological, Oncological and Anatomo-Pathological Science - UOC of Radiotherapy, Viale Regina Elena, Roma 324 00161, Italy

**Keywords:** Buschke-Löwenstein tumor, Human papillomavirus, Giant condyloma acuminatum, Chemo-radiation therapy, Wide local excision

## Abstract

Treatment of anorectal Buschke-Löwenstein tumor (BLT) with squamous cell carcinoma (SCC) transformation is not univocal given the rarity of the disease. BLT is characterized by its large size and tendency to infiltrate into underlying tissues. Malignant transformation can occur and it is important to identify the presence of neoplastic foci to decide the proper treatment. Our aim was to assess the effectiveness of neo-adjuvant chemo-radiation therapy (CRT) and local excision in order to avoid abdomino-perineal resection (APR). Three cases of anorectal BLT with SCC transformation are presented. All patients were HIV positive and treated with antiretroviral drugs. They underwent preoperative endoanal ultrasound, biopsies, total body tomography and anal brushing. Treatment consisted of neo-adjuvant chemo-radiation therapy (45 Gy to the pelvis plus a boost with 14.40 Gy to the primary tumor for a total of 59.40 Gy, and mitomycin-C in bolus on the first day, plus 5-fluorouracil by continuous infusion in the first and in the sixth week) and subsequent local surgical excision. During the follow-up, patients were subjected to the same preoperative diagnostic investigations and high resolution anoscopy. All patients showed a complete regression of the lesion after CRT and were treated by local surgical excision, thus avoiding permanent colostomy. In conclusion neo-adjuvant chemo-radiation therapy with local surgical excision could be considered an effective therapy in the treatment of anorectal BLT with SCC transformation to avoid APR.

## Background

The Buschke-Löwenstein tumor (BLT) or giant condyloma acuminatum (GCA) is a large, exophytic, cauliflower lesion of the anogenital region, correlated to HPV infection [1]. It is histologically benign and when it is diagnosed at an early stage can be treated with wide local excision [2,3]. Some authors believe that the biological behavior of the BLT is an intermediate form between condyloma acuminatum and squamous cell carcinoma [4]. Malignant transformation in squamous cell carcinoma (SCC) can occur and if not detected early it is no longer distinguishable from an invasive carcinoma [5]. There are different treatments for BLT including local surgical excision, cryotherapy or topical podophyllin [6]. In the presence of foci of SCC, therapy is still controversial. We present three cases of BLT with SCC transformation treated with chemo-radiation therapy and local surgical excision.

## Case presentation

### Case one

**Figure 1:**
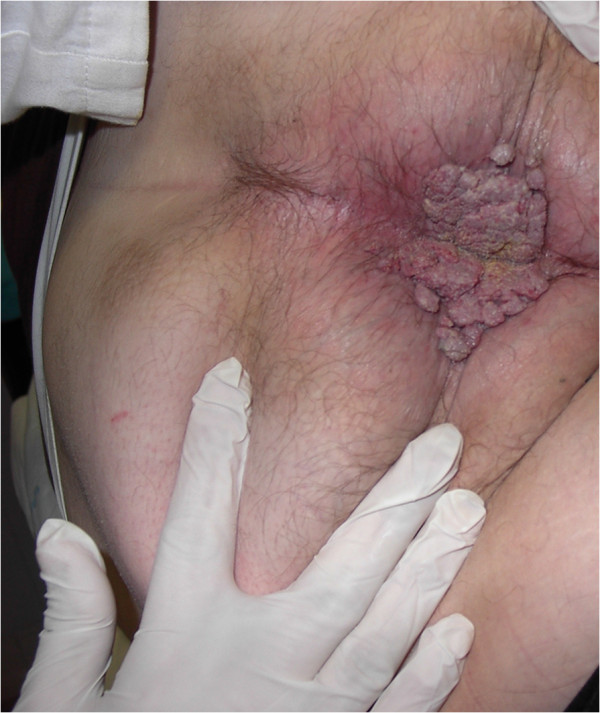
**Preoperative appearance of the patient with a perianal cauliflower-like tumor.** The lesion appears exophytic and infiltrates the perianal tissue.

**Figure 2:**
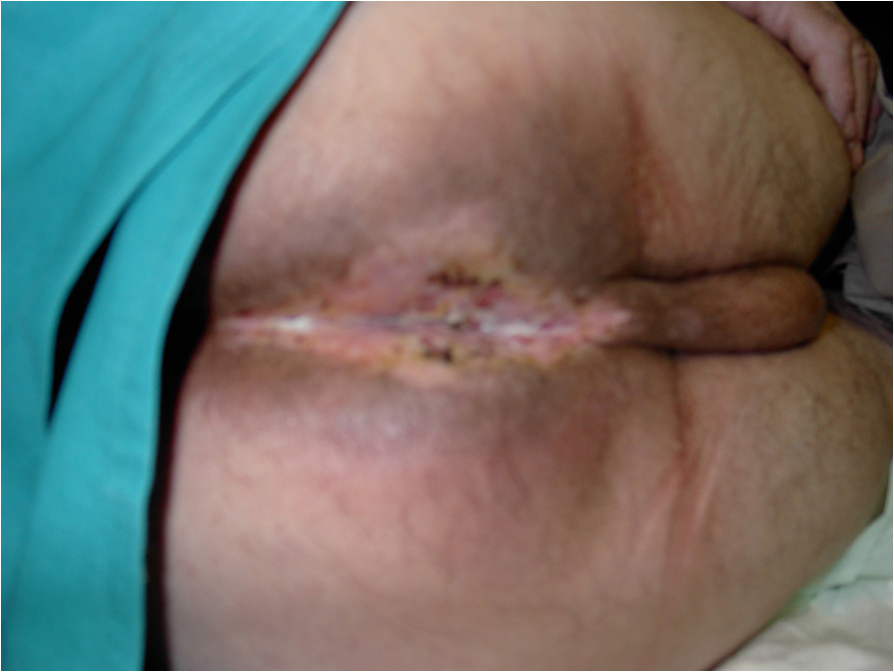
**Perineum after chemo-radiation therapy and local excision.** The lesion has completely disappeared after chemo-radiation and local excision was subsequently performed.

A 43-year-old homosexual and HIV positive man was admitted for a large perianal lesion that had given anal discomfort for two months (Figure 1). Endoanal ultrasound examination (EUS) showed anal canal invasion (uT_1_N_0_). Histological examination on multiple biopsies specimens showed a characteristic GCA with SCC transformation. PCR performed on the anal brushing revealed the presence of HPV-6. The patient submitted to chemo-radiation therapy according to the following scheme: 45 Gy (1.8 Gy/fr) to the pelvis plus a boost with 14.40 Gy (1.8 Gy/fr) to the primary tumor for a total of 59.4 Gy, and mitomycin-C (10 mg/m^2^) in a single dose on first day, plus 5-fluorouracil (750 mg/m^2^) by continuous infusion in the first and in the sixth week. He tolerated the treatment well. The tumor disappeared completely after treatment and perianal and endoanal local excisions were performed (Figure 2). Histological examination ruled out the presence of GCA and cancer. The patient underwent EUS and high resolution anoscopy (HRA) twice a year and, after three years, is disease free.

### Case two

**Figure 3:**
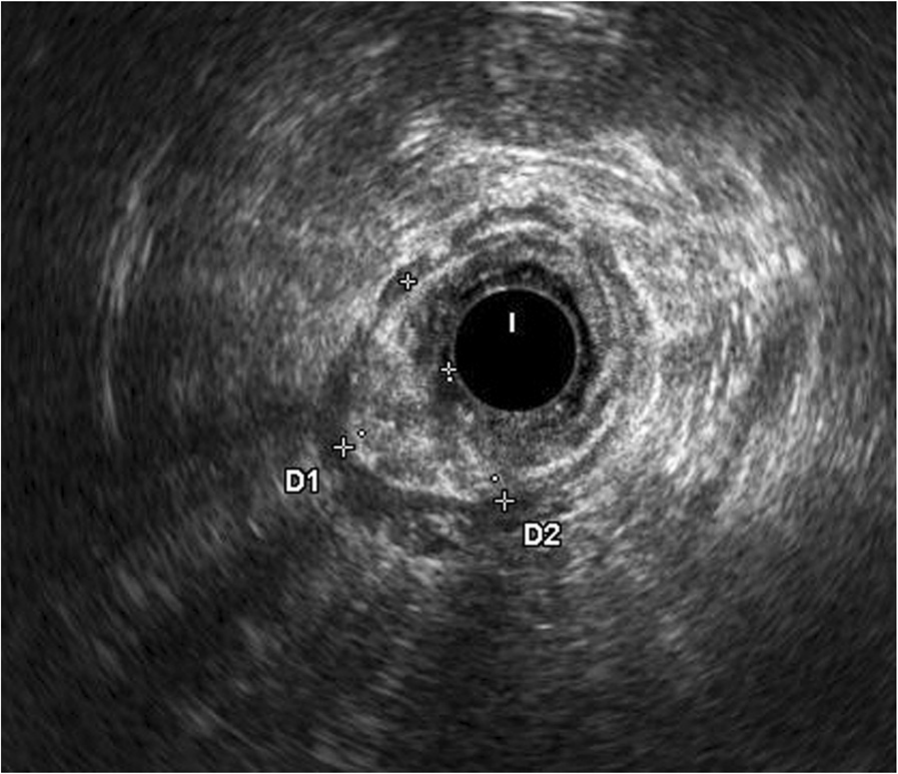
**Endoanal ultrasound (US) showing the tumor invasion of the anal canal.** Endoanal US allowed completion of the staging and showed the degree of infiltration thus staging the tumor as uT2.

**Figure 4:**
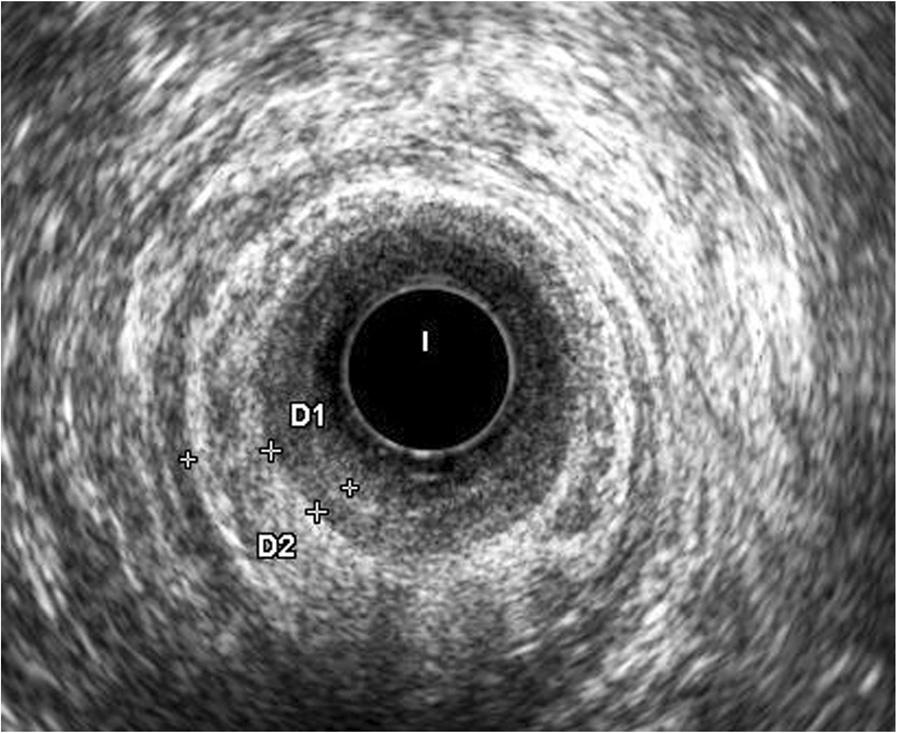
**Endoanal ultrasound (US) after treatment.** It is evident a complete regression of the anal tumor.

A 56-year-old homosexual HIV positive man was admitted complaining of anal pain and bleeding, mucopurulent perianal discharge and severe incontinence for six months due to a large perianal ulcerated lesion. EUS showed invasion of the anal canal by the tumor (uT2) (Figure 3) and histologic examination on multiple endoanal and perianal biopsies revealed the presence of GCA and foci of SCC. Pathological inguinal lymph nodes were detected by ultrasound examination (uN_1_). PCR performed on the anal brushing revealed the presence of HPV-6. The patient underwent a temporary colostomy for severe incontinence and anal fistulas and submitted to chemo-radiotherapy according to the following scheme: 45 Gy (1.8 Gy/fr) to the pelvis plus a boost with 14.40 Gy (1.8 Gy/fr) to the primary tumor for a total of 59.4 Gy, and mitomycin-C (10 mg/m^2^) in a single dose on first day plus 5-fluorouracil (750 mg/m^2^) by continuous infusion in the first and in the sixth week. He tolerated the treatment well. The perianal GCA did not disappear completely after treatment, and was removed by wide local excision (Figure 4). Histological examination ruled out the presence of cancer and the patient then underwent surgery to close the colostomy. A year later the patient presented distant metastases and was subjected to further chemotherapy. He died one year later.

### Case three

A 37-year-old homosexual, HIV positive man was treated for ten years with antiretroviral therapy. He also had syphilis, scabies and molluscum contagiosum. He was previously surgically treated for penile and anal warts. He was admitted complaining of anal itching and pain, a foul smell and problems with defecation due to a massive swelling (12 cm × 15 cm) in the perianal region. EUS highlighted that the lesion also extended into the anal canal (uT_1_N_0_). Histological examination detected the characteristic feature of the GCA and foci of SCC in the endoanal lesion. PCR performed on the anal brushing revealed the presence of HPV-18. A computed tomography confirmed the EUS and ruled out the presence of pathological lymph nodes and distant metastasis. The patient underwent chemo-radiotherapy with the following scheme: 45 Gy (1.8 Gy/fr) to the pelvis plus a boost with 14.40 Gy (1.8 Gy/fr) to the primary tumor for a total of 59.4 Gy and mitomycin-C (10 mg/m^2^) in bolus on first day plus 5-fluorouracil (750 mg/m^2^) by continuous infusion in the first and in the sixth week. He tolerated the treatment well. The perianal tumor disappeared completely after treatment, while a small lesion persisted inside the anal canal was removed by local excision. Histological examination ruled out the presence of GCA and cancer showing only fibrous tissue. The patient underwent EUS and HRA twice a year and computer tomography (CT) scan once a year. After three years, he is disease free.

### Discussion

Giant condyloma acuminatum was first described by Buschke and Löwenstein as a lesion of the penis in 1925 [7]. Subsequently, it has been observed in the anorectal and perianal regions [8]. It is a large, exophytic lesion, slow growing and with a tendency to relapse. A high incidence of GCA has been reported in the homo and bisexual populations and recurrent aggressive GCA has been reported in HIV positive patients [9]. All our cases were homosexual and HIV positive and treated with antiretroviral drugs. As regards its etiology, the HPV-6 and HPV-11 are the most common types identified but HPV-16 and HPV-18 are also detected especially in cases with foci of SCC [8,10]. In our patients, we identified HPV-6 in two patients and HPV-18 in one patient. Especially in HIV patients, the transformation in SCC should be suspected even in the presence of a strain not considered carcinogenic. The histological features are similar to those of condyloma acuminatum but remarkable thickening of the stratum corneum, marked papillary proliferation and the tendency to invade deeply are present [5]. In the past, BLT was not considered a malignant lesion but a transformation in squamous cell carcinoma can occur after an average of five years [3]. Careful histological examination is necessary to rule out the presence of SCC [5]. In a 2001 systematic review of the literature, Trombetta *et al*. found the presence of neoplastic foci in 23 of 52 patients undergoing surgery for GCA [11]. GCA differs from SCC in that it demonstrates an intact basement membrane, infrequent mitosis, absence of metastases and the tendency to displace deep tissues rather than to infiltrate. When malignant transformation is not detected early, it is no longer distinguishable from an invasive carcinoma. Topical applications such as podophyllin and immunotherapy have been used in the treatment of CGA but without success [6]. Wide local excision or abdomino-perineal resection (APR) are considered the treatment of choice of GCA depending on local extension of the lesion and the involvement of the anal canal, but Chu reported a recurrence rate of 50% after radical surgery alone [3]. In cases of GCA, an accurate preoperative study is indispensable as malignant transformation, not always diagnosed before surgery, was found in a high percentage. In 42 cases of anorectal GCA reported in the literature, malignant transformation was observed in 52% of the patients [3]. Creasman, in another review, observed malignant transformation in 30% of cases [12]. Thus, we perform EUS and CT to assess the exact extent of the disease and multiple biopsies to detect neoplastic transformation. In the presence of foci of SCC, we believe it advisable to refer patients to radio-chemotherapy, using the same protocols adopted in epidermoid cancer of the anus [13-15]. Two months after the end of treatment, we reevaluate the patients with endoanal ultrasound, total body CT scan, endoscopy and HRA. In our patients, the histological examination performed on a wide local excision has allowed us to exclude the presence of GCA and neoplastic foci which would indicate a need for APR. Butler *et al.* reported a case of anal GCA invading the perineum and pelvis, with foci of SCC treated by fecal diversion and chemo-radiation. APR was then performed with no evidence of disease in the resected specimen [16]. We believe that APR is indicated only in non-responding patients or in the presence of a large residual tumor; in all other cases we consider adequate local excision. In all our patients with extensive involvement of the perianal region and the anal canal, neo-adjuvant radio-chemotherapy has allowed the preservation of the anal sphincters and complete cure of the local disease. The patient who died two years after surgical treatment from distant metastases had more advanced disease at diagnosis, with involvement of the inguinal lymph nodes. Despite this, neo-adjuvant therapy allowed a loco-regional control of the disease, thus avoiding colostomy. We believe that the patients treated with this multimodal therapy should undergo careful clinical and instrumental follow-up to identify local recurrence in the early stages when it can still be susceptible to further surgical treatment. Prolonged follow-up is also indicated because the majority of patients with GCA are homosexual and HIV positive and, therefore, may undergo reinfection with HPV and development of new cancer of the anus [17].

## Conclusion

Neo-adjuvant chemo-radiation therapy with local surgical excision could be considered an effective therapy in the treatment of anorectal BLT with SCC transformation to avoid APR.

## Consent

Written informed consent was obtained from the patients for publication of this case report and any accompanying images. A copy of the written consent is available for review by the Editor-in-Chief of this journal.

## Abbreviations

APR: Abdomino-perineal resection; BLT: Buschke-Löwenstein tumor; CT: Computer tomography; EUS: Endoanal ultrasound; GCA: Giant condyloma acuminatum; HPV: Human papillomavirus; HRA: High resolution anoscopy; PCR: Polymerase chain reaction; SCC: Squamous cell carcinoma; US: Ultrasound.

## Competing interests

The authors declare that they have no competing interests.

## Authors’ contribution

MI has devised the treatment strategy, treated patients with surgery and coordinated the various experimenters. AI assisted with the surgery, researched the literature and drafted the manuscript. GB made the endoanal diagnosis and performed the images. CMM assisted with the surgery. PG assisted with the surgery and reviewed the literature. AC made the histological diagnosis. GD followed patients for antiretroviral therapy. CB, DM, FD, VT treated patients with chemo-radiation therapy. All authors read and approved the final manuscript.
